# Supplementary material for: DMRforPairs: identifying Differentially Methylated Regions between unique samples using array based methylation profiles
Source: BMC Bioinformatics. 2014 May 15;15:141. doi: 10.1186/1471-2105-15-141 (PMC4046028; doi:10.1186/1471-2105-15-141)
Supplement: Additional file 2 — DMRforPairs output for the comparison of A431-MCF7 and NA17018-NA17105. Please start from the HTML files in each folder. Available via the BMC Bioinformatics website. [file 1471-2105-15-141-S2.zip › 1394847754114233_MOESM2_ESM/A431_MCF7/figures/31730.pdf]

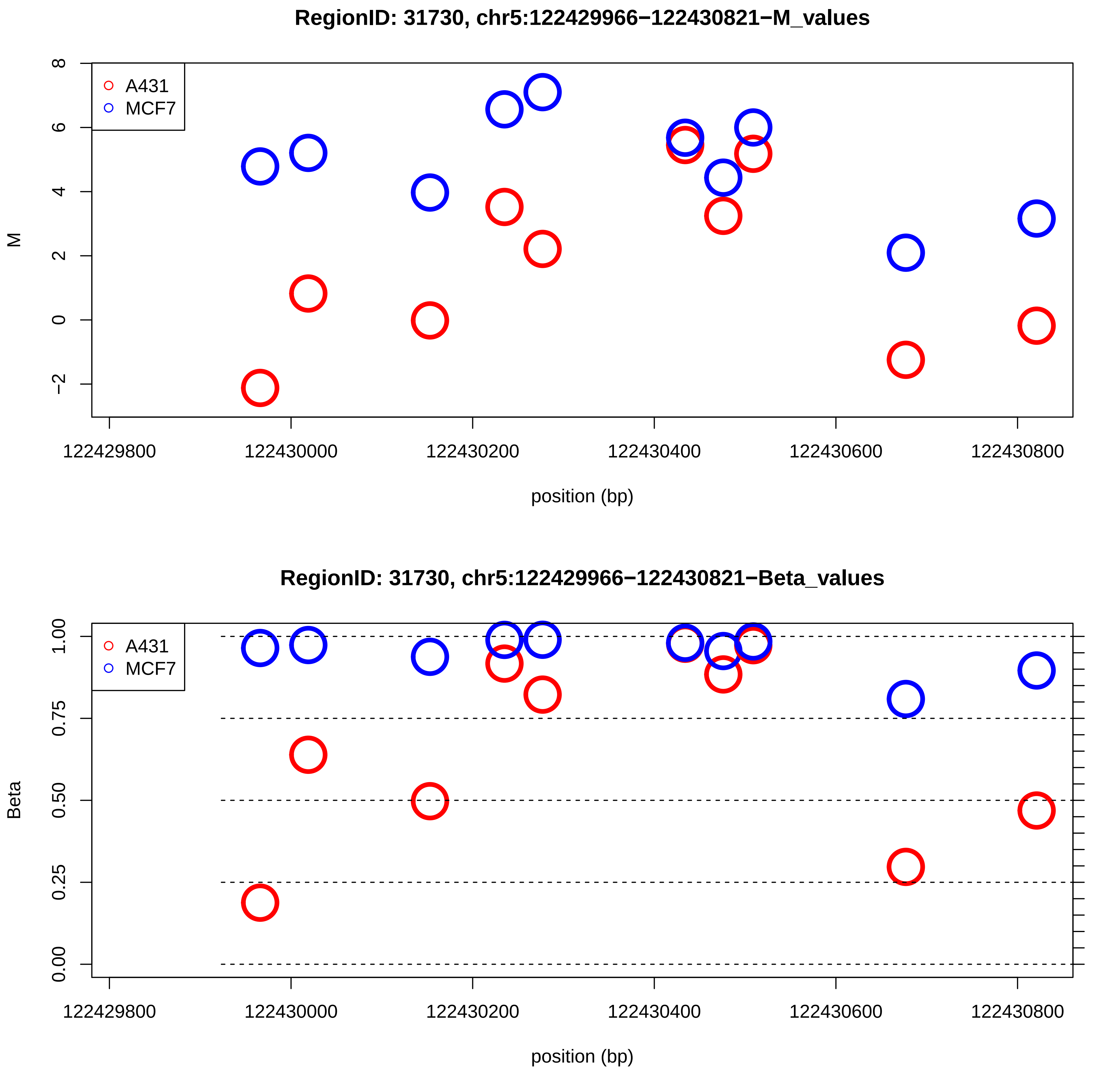

RegionID: 31730, chr5:122429966−122430821−M_values
A431
MCF7
M
−2
0
2
4
6
8
122429800
122430000
122430200
122430400
122430600
122430800
position (bp)
RegionID: 31730, chr5:122429966−122430821−Beta_values
A431
MCF7
Beta
0.00
0.25
0.50
0.75
1.00
122429800
122430000
122430200
122430400
122430600
122430800
position (bp)

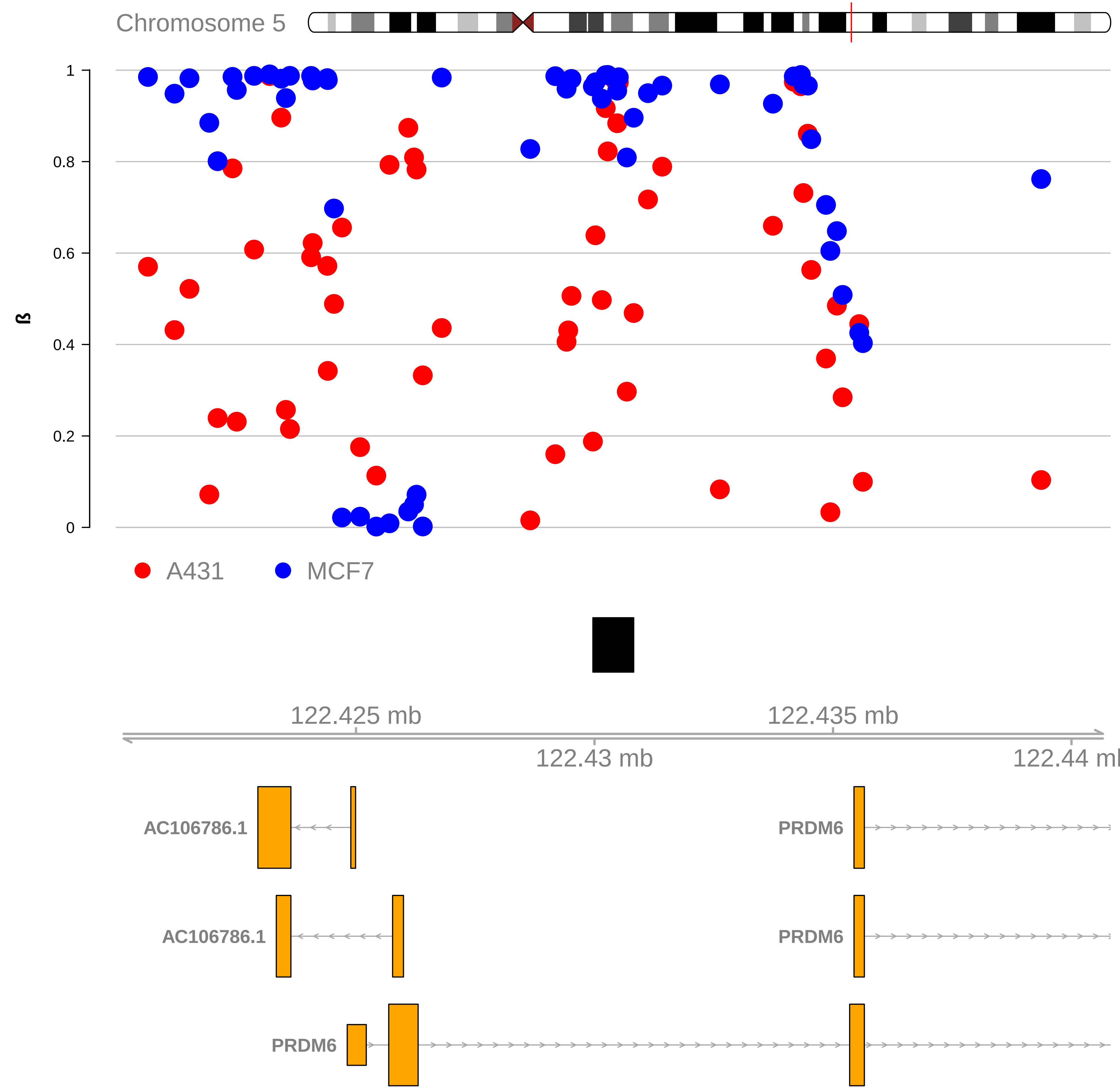

Chromosome 5
1
0.8
0.6
0.4
0.2
0
ß
A431
MCF7
122.425 mb
122.435 mb
122.43 mb
122.44 mb
AC106786.1
PRDM6
AC106786.1
PRDM6
PRDM6
